# Investigation of Optoelectronic Functional Crystals: Crystal Growth, Properties and Applications

**DOI:** 10.3390/ma16216871

**Published:** 2023-10-26

**Authors:** Lihong Shi, Wenbo Yan

**Affiliations:** 1 Department of Physics, Tianjin Chengjian University, Tianjin 300384, China; 2 State Key Laboratory of Reliability and Intelligence of Electrical Equipment, School of Materials Science and Engineering, Hebei University of Technology, Tianjin 300130, China

Optoelectronic functional crystals facilitate the conversion of light and electricity, inspiring breakthroughs in the fabrication of optoelectronic devices. These contraptions play important roles in the development of microelectronics, optoelectronics, communication, aerospace, and modern military technology. There are wide range of optoelectronic functional crystal types, including piezoelectric crystals, electro-optic crystals, laser crystals, nonlinear optical crystals, and scintillation crystals. From the discovery of mineral crystals to the applications of artificial crystals, significant progress has been made in the structural characterization, property modification, growth technique development and application adaptation of optoelectronic functional crystals. In recent years, metal halide perovskite has become an ideal choice for use in photodetectors due to its adjustable bandgap, high carrier mobility, high absorption coefficient, and ease of solution processing. Perovskite-type ferrites are also promising materials for use in electrochemical devices to convert and store energy, such as solid oxide fuel cells and membrane reactors for oxygen separation from air. The accurate characterization and fine-tuning of the crystal structure are crucial for improving the detection sensitivity, response speed, energy conversion and storage efficiency of the relevant devices for both types of perovskite crystal. Third-generation semiconductor materials, represented by aluminum nitride and gallium nitride, have superior properties such as high bandgap width, high breakdown field strength, high thermal conductivity, and low energy loss.

The light-emitting diodes based on these materials show great potential in fields of air and water purification, biochemical detection, sterilization and disinfection, due to their advantages of small size, long lifespan, and adjustable emission wavelength. Lattice mismatch or thermal mismatch in the growth of semiconductor epitaxial layers can lead to the formation of dislocation defects in the crystals, thereby significantly reducing the performance of relevant devices. Introducing a buffer layer or post-growth annealing procedure can effectively suppress the formation of these defects. The effects of buffer-layer growth and post-growth annealing on the surface morphology and crystalline quality of the epitaxial layers obviously deserves systematic investigation. Organic optoelectronic functional materials have advantages of flexibility, low cost and a simple preparation process. They also demonstrate excellent performance in devices such as luminescence, solar cells, field-effect transistors, biosensors, and solid-state lasers. Organic optoelectronic functional materials are usually applied in the form of thin films, which allows for the integration of diverse organic optoelectronic devices into an advanced intelligent system based on optical communication, high-resolution imaging, and health monitoring. Developing a simple and efficient in situ growth method for organic single crystal has become a key step in the integration of these organic optoelectronic devices. Meanwhile, further studies of deep learning, computer vision, robotics and artificial intelligence, deeply involved in the intelligent optoelectronic system, are also beneficial to the final practicalization of the optoelectronic devices.

This Special Issue is issuing a call for research papers and review articles encompassing the growth, properties and potential applications of optoelectronic functional crystals:

Topics include, but are not limited to, the following area:

(1) Preparation and characterization of optoelectronic materials;
(2) First-principles study of optoelectronic materials;
(3) Photocatalytic degradation and photothermal conversion;
(4) Light emitting and infrared/X-ray imaging;
(5) Optical modulation and waveguide;
(6) Wavelength conversion and coherent optical amplification;
(7) Optofluidic manipulation and interfacial science;
(8) Deep learning and computer vision for optoelectronic applications;
(9) Robotics and artificial intelligence for optoelectronic applications;

The guest editors hope that the selected papers for this Special Issue will help scholars and researchers to advance and develop the growth, properties and applications of optoelectronic functional crystals, and also provide some valuable information and recommendations for the development of microelectronics, optoelectronics, communication, aerospace, and modern military technology.

